# Clinical, Virologic, and Epidemiologic Characteristics of Dengue Outbreak, Dar es Salaam, Tanzania, 2014

**DOI:** 10.3201/eid2205.151462

**Published:** 2016-05

**Authors:** Francesco Vairo, Leonard E.G. Mboera, Pasquale De Nardo, Ndekya M. Oriyo, Silvia Meschi, Susan F. Rumisha, Francesca Colavita, Athanas Mhina, Fabrizio Carletti, Elibariki Mwakapeje, Maria Rosaria Capobianchi, Concetta Castilletti, Antonino Di Caro, Emanuele Nicastri, Mwelecele N. Malecela, Giuseppe Ippolito

**Affiliations:** National Institute for Infectious Diseases L. Spallanzani, Rome, Italy (F. Vairo, P. De Nardo, S. Meschi, F. Colavita, F. Carletti, M.R. Capobianchi, C. Castilletti, A. Di Caro, E. Nicastri, G. Ippolito);; National Institute for Medical Research, Dar es Salaam, Tanzania (L.E.G. Mboera, N.M. Oriyo, S.F. Rumisha, A. Mhina, M.N. Malecela);; Ministry of Health and Social Welfare, Dar es Salaam (E. Mwakapeje)

**Keywords:** dengue, dengue virus, dengue virus serotype 2, DENV-2, viruses, outbreak, virology, epidemiology, Dar es Salaam, Tanzania

## Abstract

We investigated a dengue outbreak in Dar es Salaam, Tanzania, in 2014, that was caused by dengue virus (DENV) serotype 2. DENV infection was present in 101 (20.9%) of 483 patients. Patient age and location of residence were associated with infection. Seven (4.0%) of 176 patients were co-infected with malaria and DENV.

Data are scarce on seroprevalence of dengue virus (DENV) in Tanzania. Cross-sectional studies conducted during 2007–2014 indicated that DENV seroprevalence ranged from <2% to >50%, depending on geographic area and epidemiologic characteristics of patients (*1**–**6*). *Aedes aegypti* mosquitoes, the main vector of DENV, are present throughout Tanzania (*7*), and the clinical course of DENV infection is greatly affected by previous exposure to different DENV serotypes (*8*). Investigation of DENV outbreaks might serve to define circulation of different serotypes and the best strategy to manage future outbreaks.

In 2014, a large dengue outbreak occurred in Dar es Salaam, Tanzania (*8*). We report the main findings of a study conducted there during the outbreak.

## The Study

Ethical approval for the study was obtained from the National Health Research Ethics Sub-Committee of Tanzania (protocol no. NIMR/HQ/R.8a/Vol. IX/I733). Informed consent was obtained from all participants.

**Figure 1:**
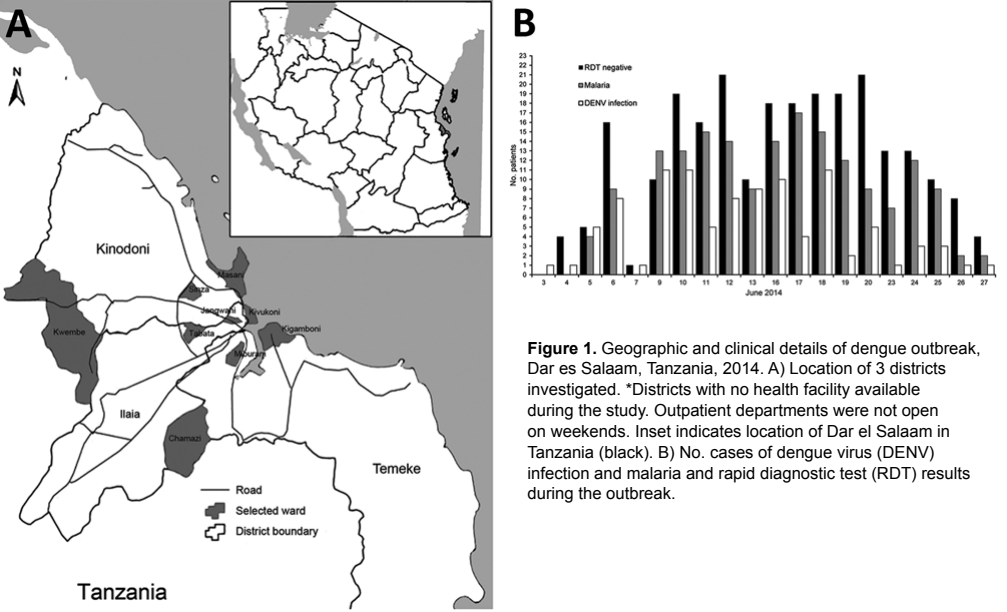
Geographic and clinical details of dengue outbreak, Dar es Salaam, Tanzania, 2014. A) Location of 3 districts investigated. *Districts with no health facility available during the study. Outpatient departments were not open on weekends. Inset indicates location of Dar el Salaam in Tanzania (black). B) No. cases of dengue virus (DENV) infection and malaria and rapid diagnostic test (RDT) results during the outbreak.

The study involved the 3 districts of Dar es Salaam (Kinondoni, Ilala, and Temeke (Figure 1, panel A). All consecutive patients with fever (temperature >37.5°C) for <7 days who came to the outpatient department of 1 of 7 selected health facilities were tested for malaria by using a malaria rapid diagnostic test (mRDT) (SD Bioline Malaria Ag. Pf/Pan Test, Bioline, Gewerbestrasse, Switzerland) and for dengue by using a DENV rapid diagnostic test (dRDT) (SD Bioline Dengue Duo; Standard Diagnostics, Inc., Gyeonggi-do, South Korea).

DENV infection was defined as a positive result for DENV nonstructural protein 1 (NS1) or IgM against DENV alone detected by dRDT. Past DENV infection was defined as IgG against DENV detected by dRDT. Malaria infection was defined as a positive result by mRDT. Warning signs and severe dengue were defined according to guidelines of the World Health Organization (*9*). A structured interview was used to collect demographic, environmental, and clinical data. Molecular analyses were conducted at the National Institute for Infectious Diseases L. Spallanzani (Rome, Italy).

**Figure 2:**
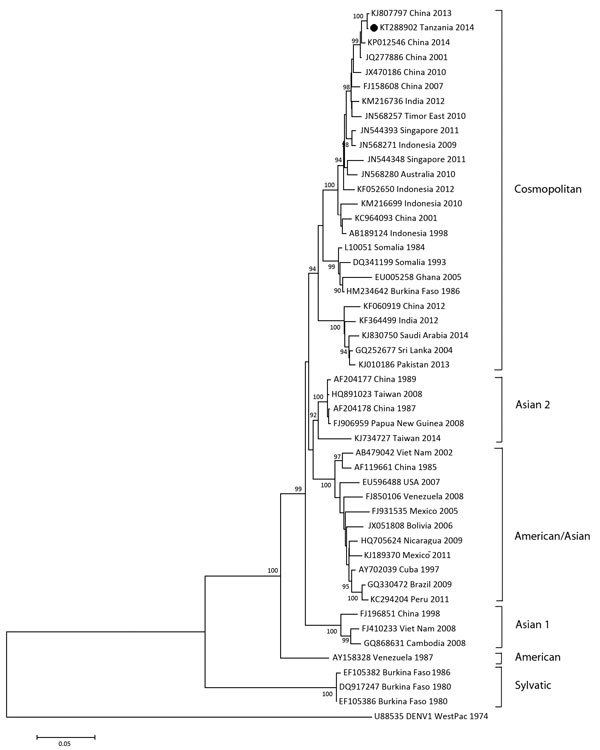
Phylogenetic analysis of complete envelope gene sequences (1,485 nt, position 937–2421 of strain KP012546) of 48 dengue virus serotype 2 (DENV2) strains representing 6 genotypes, Dar es Salaam, Tanzania, 2014. Bootstrap values (>90%) are shown at key nodes. DENV1 West Pacific was used as an outgroup. Solid circle indicates strain isolated in this study. Scale bar indicates nucleotide substitutions per site.

Virus RNA was extracted and 2 real-time reverse transcription PCRs (RT-PCRs), one specific for DENV serotypes 1, 2, and 3 and one for DENV serotype 4, were performed on dRDT-positive serum samples as described (*10**,**11*). For identification of DENV serotypes, a fragment spanning the E–NS1 gene junction was amplified as described (*12*). A complete envelope (E) gene sequence was obtained by using a One-Step RT-PCR Kit (QIAGEN, Hilden, Germany) and primers (primer sequences available on request). Sanger sequencing and phylogenetic analysis based on the nucleotide sequence of the E–NS1 region and a complete E gene sequence (Figure 2) were performed. A multiple logistic regression model with a backward procedure was used to determine a cutoff level of p = 0.10.

A total of 483 of 491 patients who came to health facilities and matched inclusion criteria were enrolled in the study. Among enrolled patients, 101 (20.9%) were positive for DENV infection, and 9 (1.9%) were positive for past DENV infection. Incidence of DENV infections peaked during mid-June and decreased toward the end of the month (Figure 1, panel B).

Univariate analysis showed that current DENV infection was associated with patient age (p<0.001), location of residence (p = 0.007), and employment status (p<0.001) (Table). Multivariate analysis showed that only age and location of residence were independently associated with current DENV infection. The risk for infection for patients >15 years of age was twice that for patients <15 years of age (odds ratio 2.54, 95% CI 1.10–5.87; p = 0.029). Patients who lived in Kinondoni were twice as likely to have a DENV infection than patients who lived in Temeke (odds ratio 2.83, 95% CI 1.44–5.56; p = 0.002).

**Table:** Characteristics of 483 patients tested for DENV infection during dengue outbreak, Dar es Salaam, Tanzania, 2014*

Characteristic	Current dengue infection, n = 101	No dengue, n = 382	Total, n = 483	p value
Sex				0.067†
M	65 (64.4)	207 (54.2)	272 (56.3)	NA
F	36 (35.6)	175 (45.8)	208 (43.7)	NA
Median age, y (IQR)	28 (21–37)	24 (13–35)	25 (14–36)	<0.001‡
Age, y, n = 481				<0.001†
<15	12 (11.9)	117 (30.8)	129 (26.8)	NA
>15	89 (88.1)	263 (69.2)	352 (73.2)	NA
District				0.007†
Temeke	18 (17.8)	123 (32.2)	141 (29.2)	NA
Ilala	34 (33.7)	128 (33.5)	162 (33.5)	NA
Kinondoni	49 (48.5)	131 (34.3)	180 (37.3)	NA
Employed, n = 455	66 (69.5)	169 (46.9)	235 (51.7)	<0.001†
Water storage, n = 476	43 (43.9)	185 (48.9)	228 (47.9)	0.371†
Resting water, n = 481	48 (48.5)	179 (46.9)	227 (47.2)	0.773†
Persons in household, n = 474				0.284†
1–3	33 (33.3)	118 (31.5)	151 (31.9)	NA
4–6	41 (41.4)	185 (49.3)	226 (47.7)	NA
>7	25 (19.2)	72 (19.2)	97 (20.5)	NA
Bed net use, n = 479	75 (75)	313 (82.6)	388 (81)	0.085†
Insecticide spraying in home, n = 479	20 (20)	87 (21)	107 (22.3)	0.528†

*Values are no. (%) except as indicated. DENV, dengue virus; NA, not applicable; IQR, interquartile range. †By χ^2^ test.  ‡By Wilcoxon rank-sum test.

Only joint and muscle pain were associated with DENV infection (p<0.001). Warning signs were more frequent in patients with DENV infection (42/101, 41.6%; p = 0.006). Three patients met criteria for severe dengue. Patients with DENV infection were more likely to be hospitalized (p<0.001) and to have used antimalarial drugs (p = 0.025). A total of 176 (34.6%) patients had a positive result for malaria by mRDT. Of these patients, 7 (4.0%) were co-infected with DENV (positive result for NS1), and 14 (8.0%) had a recent DENV–malaria coinfection (IgM or IgM/IgG positive). Sixty-two patients with DENV infection had positive results by PCR.

Sequence analysis of NS1–E junction gene fragments of DENV was performed for 8 randomly selected RT-PCR–positive samples (GenBank accession nos. KT288895–KT288902). Phylogenetic analysis of all sequences grouped them in a monophyletic cluster in the DENV serotype 2 cosmopolitan genotype. Further sequencing of the complete E gene was performed for 1 isolate (GenBank accession no. KT288902). Clustering with the DENV serotype 2 cosmopolitan genotype was confirmed (Figure 2). This analysis showed similarity of DENV in Tanzania with those from Asia isolated since 2001 and a strong phylogenetic relationship (99.8% identity) with a DENV variant isolated in Guangzhou, China, in 2013 (GenBank accession no. KJ807797).

## Conclusions

We report DENV serotype 2 (Cosmopolitan genotype) as the causative agent of the dengue outbreak in Dar es Salaam in 2014. Sequence analysis showed that this virus from Tanzania had a strong phylogenetic relationship with virus strains from China, India, East Timor, and Singapore. These results indicate that the virus could have been recently introduced into Tanzania by travelers from Asia. This hypothesis is supported by the phylogenetic relationship with sequences obtained from isolates in Asia since 2001 and by reports of DENV-3 detection in the previous years in Zanzibar (*4**,**5*).

The incidence of DENV infection in Dar es Salaam (20.9%) is higher than incidences reported in recent studies of household participants in Angola (9% of recent infections) (*13*) and Kenya (13% of recent/current infections). This discordance might be related to different designs of these 2 studies. Our study was conducted with febrile patients attending health facilities and not in households. Moreover, the dRDT used in our study has a lower specificity than that of tests used in Angola and Kenya.

The higher incidence of DENV infection among elderly patients is not consistent with recent data for other disease-endemic regions (*13*) and might be related to possible recent introduction of the DENV serotype 2 in the area. The higher incidence reported in Kinondoni District could be related to the urban nature of the district.

As reported in Africa (*13*,*14*), no environmental or behavioral factors were associated with DENV infection. Malaria and DENV co-infection was present in 4% of patients, a find similar to that in a recent study in Ghana, where possible co-infection in was reported in 3.2% of children (*15*). As reported in Kenya (*14*), the high use rate (33.3%) for antimalaria treatment in patients with DENV infection during the current outbreak suggests a high level of underrecognition of dengue.

External validity of our results should be evaluated according to potential limitations of the study. First, the study used passive recruitment, which might have resulted in potential selection bias (only sicker patients who came to selected health facilities were analyzed). Second, the study had virtually no follow-up, which precluded any inferences about clinical outcomes of patients with severe dengue.

Despite these limitations, our study provides useful information on an underreported disease and on the molecular epidemiology of largest dengue outbreak reported in Tanzania. Results of our study can improve awareness of healthcare providers and demonstrate the feasibility of interventions to enhance diagnostic testing capabilities and specific surveillance systems. Resurgence of dengue in Tanzania should prompt implementation of population-based studies on differential diagnosis of acute febrile illness and surveillance systems based on syndromic approaches.
